# MineLoC: A Rapid Production of Lab-on-a-Chip Biosensors Using 3D Printer and the Sandbox Game, Minecraft

**DOI:** 10.3390/s18061896

**Published:** 2018-06-10

**Authors:** Kyukwang Kim, Hyeongkeun Kim, Seunggyu Kim, Jessie S. Jeon

**Affiliations:** 1 Robotics Program, Korea Advanced Institute of Science and Technology, 291 Daehak-ro, Daejeon 34141, Korea; kkim0214@kaist.ac.kr; 2 Department of Mechanical Engineering, Korea Advanced Institute of Science and Technology, 291 Daehak-ro, Daejeon 34141, Korea; hkkim1227@kaist.ac.kr (H.K.); ksg5825@kaist.ac.kr (S.K.); 3 KAIST Institute for Health Science and Technology, 291 Daehak-ro, Daejeon 34141, Korea

**Keywords:** Lab-on-a-Chip, 3D printing, additive manufacturing, microfluidics, solid modeling

## Abstract

Here, MineLoC is described as a pipeline developed to generate 3D printable models of master templates for Lab-on-a-Chip (LoC) by using a popular multi-player sandbox game “Minecraft”. The user can draw a simple diagram describing the channels and chambers of the Lab-on-a-Chip devices with pre-registered color codes which indicate the height of the generated structure. MineLoC converts the diagram into large chunks of blocks (equal sized cube units composing every object in the game) in the game world. The user and co-workers can simultaneously access the game and edit, modify, or review, which is a feature not generally supported by conventional design software. Once the review is complete, the resultant structure can be exported into a stereolithography (STL) file which can be used in additive manufacturing. Then, the Lab-on-a-Chip device can be fabricated by the standard protocol to produce a Lab-on-a-Chip. The simple polydimethylsiloxane (PDMS) device for the bacterial growth measurement used in the previous research was copied by the proposed method. The error calculation by a 3D model comparison showed an accuracy of 86%. It is anticipated that this work will facilitate more use of 3D printer-based Lab-on-a-Chip fabrication, which greatly lowers the entry barrier in the field of Lab-on-a-Chip research.

## 1. Introduction

The development of Lab-on-a-Chip (LoC) technology launched a new era in biological and chemical analysis systems with its advantages in the usage of small volumes, low-cost, compactness and more. However, the conventional soft lithography-based technique for LoC manufacturing requires specialized infrastructures and skills that hinder the accessibility of potential users.

Additive manufacturing (AM), commonly referred to as “3D printing”, is a layer-by-layer adding manufacturing process which includes seven subgroup technologies classified by ASTM/ISO (ASTM52900-15) [1]. It was originally used for rapid prototyping in manufacturing industries; however, recent advances of related technologies have released affordable material extrusion or Vat photopolymerization “3D printers” to markets which can be easily purchased by the general public. Open-source hardware and related movements such as FabLab [2] have made it much easier for students or developers to access 3D printers than before. Rather than using lithography, with 3D printers, the templates for polydimethylsiloxane (PDMS) can be directly printed, allowing for low-cost fabrication and faster feedback. Several researchers successfully demonstrated the feasibility of the approach. Comina et al. [3] used a 3D printer to make a master template for molding PDMS devices. Kitson et al. and Symes et al. [4,5] proposed a 3D printed device for a chemical reactor. Costa et al. [6] and Lind et al. [7] successfully recreated 3D architecture of vascular and muscular tissue, respectively. Other 3D-printed designs also succeeded in lowering the cost of particle/cell detection [8,9], developing micro-scale culture devices [10,11], and achieving minimally invasive biopsy via bio-inspired conformal microfluidic device directly interfacing with the whole organ [12].

Despite the obvious advantages of using 3D printers, the entry barrier of additive manufacturing hinders its widespread application. The knowledge of the design software and tools for creating 3D models, a requirement for using 3D printers, is not common in the general public or researchers who would benefit by using LoC devices. The Object-Oriented Mechanics Library (OOML) and Open Solid 3D Computer Aided Design Modeller (OpenSCAD) [13,14] provide programmable interfaces, but knowledge and skills about 3D geometry are still required. To this end, a few researchers have attempted to create easy-to-use 3D modeling software for additive manufacturing, but with limited success.

Here, MineLoC is described as a pipeline to generate directly printable 3D models of the PDMS curing master template more easily by using a popular multi-player voxel sandbox game “Minecraft”. The sandbox style game has all the contents of the virtual world consisting of equal-sized cube units called “blocks”. Users can build their own constructions by adding and breaking blocks, which is like playing with toy blocks in the real world. Minecraft is an extremely popular sandbox game, whose registered users recently surpassed 100 million. Researchers have used this game for teaching scientific concepts [15] and developing Artificial Intelligence (AI) for games [16]. They proposed using Minecraft since it allows high utility with a simple and easy interface. Recent open-source software also provides methods to extract constructions in the game world into the 3D models which can be printed from 3D-printers. The authors have focused on these merits of the sandbox game; individuals or research groups with no experience in 3D modeling or geometry can easily build, share, and co-operate on their own models by playing the game. The block-by-block building process can be labor intensive, but this problem can be solved by using the “mods” (plugins that modify games) which help edit large chunks of blocks. An entertainment factor of the sandbox game is also featured. Recent research results show a combination of a game with an academic purpose (known as gamification) can greatly induce the participation and interests of the users. A game named “Foldit” allows multiple game players to calculate protein 3D structures in an online environment and predict the unknown protein structures [17]. The game “Sea Hero Quest” induces participation of the subjects to generate human memory and cognition experimental data which can be used for brain disease research [18]. Though some free or easy modeling software already exists, the proposed method has comparative advantages since it could lead interest from the new users or students. Similarly, the project developing LEGO blocks with mounted Lab-on-a-Chip devices is also introduced [19] to help educators and researchers.

**Figure 1:**
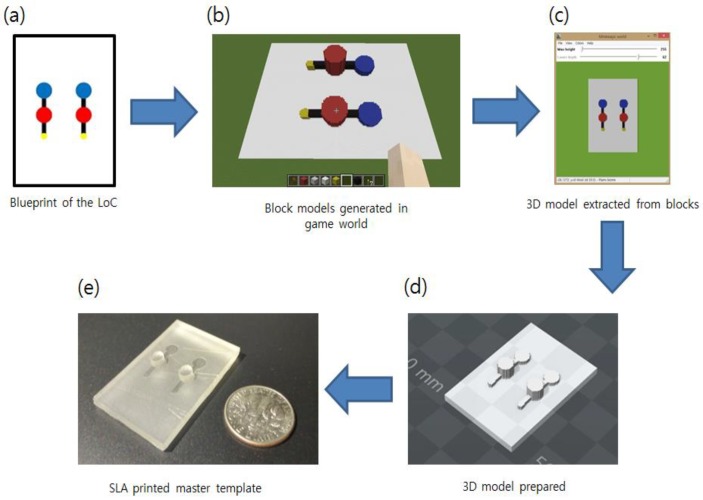
The processing flow of the MineLoC: (**a**) blueprint image with the different colors describes the function of the block; (**b**) block structure in the game world is generated based on the blueprint; (**c**) block structure is extracted using a freeware program; (**d**) 3D model is prepared for additive manufacturing; and (**e**) master template is printed using 3D printer.

We have developed JavaScript-based scripts which convert colored images into the block structures in the sandbox game. One can draw a simple diagram describing the channels and chambers of the PDMS devices and easily convert it to large chunks in the game world rather than building every block element manually or drawing a 3D model with other software. Subsequent to modifying the auto-generated blocks, the constructions can be converted to 3D printable files and printed. Overall flow of the proposed pipeline is shown in Figure 1. The outputs of MineLoC with ones made by a conventional approach have been compared as well. Additional examples and applications are introduced.

## 2. Materials and Methods

### 2.1. Chip Blueprint Preparation

A colored blueprint image representing the top-view of the master template was required. Each color of the parts represented pre-defined parts: blue represented the large in/outlet channel for the tubing, yellow indicated a small in/outlet for a syringe, and red meant a large culture chamber. White parts did not generate specific structures and only base blocks were made. Any type of drawing tool was acceptable. Image resizing to about 150-pixel height/width was required due to the memory limitation of the sandbox game. To improve the quality of the resized images, Portable Network Graphics (PNG) format was recommended for the generation of the original blueprint image.

Each pixel of the blueprint image was transformed into the single “block” in the game world. During the extraction of the blocks into the STL 3D file, each size of the block was determined (Further described in the next section). To illustrate, if the researcher wants to build a 3 cm size PDMS mold and the image is 150 pixels, block size can be determined by calculating the ratio of pixel number and desired size (0.2 mm per each block for this case). The final model scale can be adjusted by the model pre-processing software provided with the 3D printer. The minimum resolution also depends on the 3D printer used. Obtaining the conversion ratio allowed the size of each component or structure on the chip to be determined. To explain, if the black channel is 10 pixels wide, it would become a 2 mm wide channel after 3D printing if the same ratio of the above example was applied. When drawing a small-scale image is difficult, one can draw a large image with the same ratio and resize it before the conversion. The researcher can generate models with both extemporaneous drawings and elaborately designed schematics by using this technique.

### 2.2. Virtual Environment and Server Setup

The blueprint was loaded into the CraftBukkit powered Minecraft Server. A JavaScript-based modification to the Minecraft program was developed to generate a world according to the given blueprint. The scale of the blueprint can be set by the user (e.g., 1 mm per pixel). Once the world was generated, multiple users could access and modify the structure simultaneously. Prior to additive manufacturing, an STL file was generated by the Minecraft MineWays plugin. In other words, the virtual world, generated from the blueprint file and served in MineLoC server, was modified into intended design and downloaded into 3D-printable STL file. For details regarding the setup and deployment of MineLoC server, as well as the operations of MineLoC for users connected to the virtual world, refer to Appendix A and Appendix B, respectively. The program, as well as complete instructions, is available on GitHub (https://github.com/W5-KAIST/MineLoc).

### 2.3. 3D-Printing and Chip Fabrication

The Stereolithography (SLA) method-based 3D printer was used for the printing of the modeled master template as the surface finish of SLA printed outputs are the finest. The Nobel 1.0 printer (XYZprinting, Inc., San Diego, CA, USA) which supports 300 microns of *X*/*Y* axis resolution and 25 microns of *Z* axis resolution was used. Company provided resin which contains 35–50% urethane acrylate, 40–50% acrylic monomer, and photoinitiator was used as a printing material with the layer height option of 0.025 mm. The printed mold was washed with isopropyl alcohol to clean the surface and dried for 24 h. No supports were printed as the device is flat shaped without a floating structure. PDMS LoC devices were fabricated from the master templates according to the previously developed protocols. The 3D printed structure was used to fabricate LoC by a standardized protocol [20].

### 2.4. Bacterial Cell Culture on PDMS Chips

*Pseudomonas aeruginosa* was used as a test strain. Thawed stock was injected into the 40 mL Tryptic Soy Broth (TSB) and overnight incubation was done at 36 °C. To remove glycerol added during freezing, 10 μL bacteria cultured TSB were inoculated into the clean Luria–Bertani (LB) broth. A PDMS device was filled with the inoculated LB medium and cultured in the same condition.

## 3. Results and Discussion

### 3.1. Generated Model Comparison with the Conventional Method

To compare the performance of MineLoC against conventional design tools, the authors created two template models, one with a conventional design tool and another by MineLoC based on the same blueprint.

**Figure 2:**
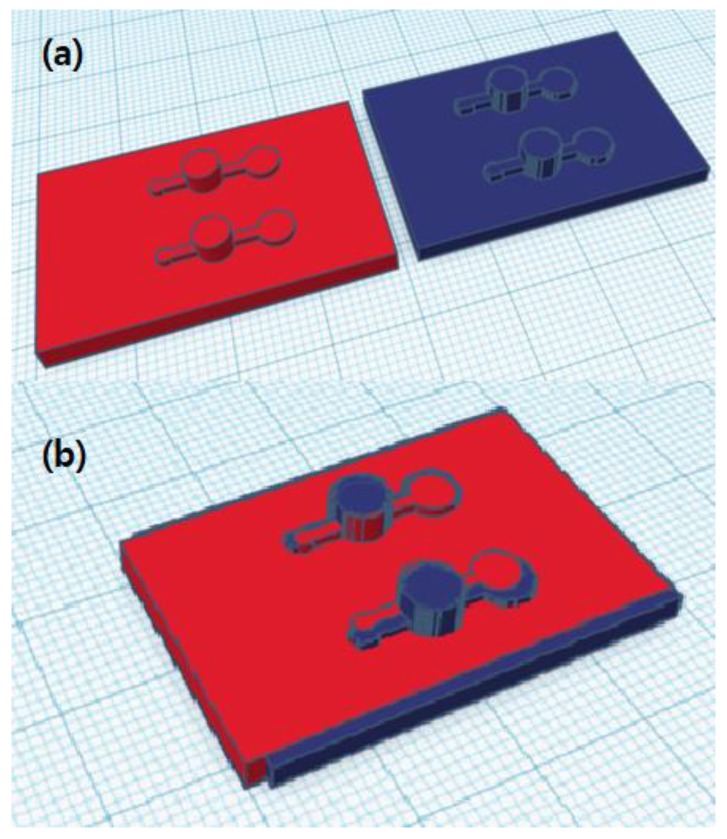
Comparison between template model generated by conventional design tools and MineLoC generated model: (**a**) side-by-side comparison; and (**b**) overlapped comparison. The red model was generated by the conventional software and the blue model was generated by the proposed method.

**Figure 3:**
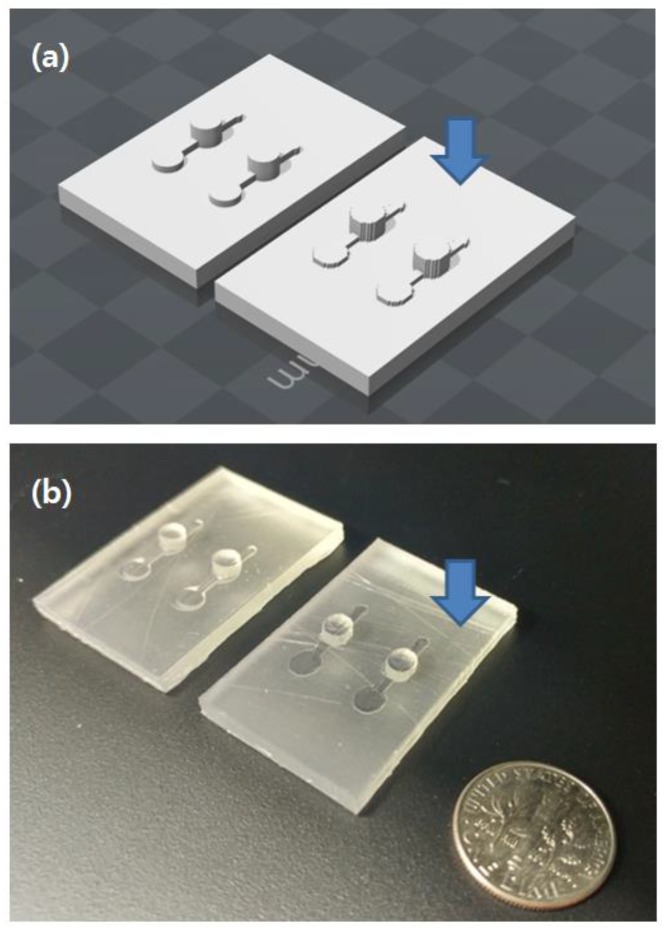
Comparison between MineLoC and conventional design tool (SolidWorks). Master templates generated using MineLoC method is marked with an arrow. (**a**) 3D printer-readable file converted models generated by the proposed method and the conventional method; and (**b**) SLA 3D printed master templates.

Figure 2 shows the comparison between two 3D-printed outputs: one modeled by the commercial software and the other by the proposed methods. Figure 2b, where two models are overlapped for comparison, the curved edge part of the blue model (designed by MineLoC) seemed slightly rough compared to the red model (conventionally designed) due to a resolution problem, but without further noticeable differences. Small position coordinate errors due to the usage of eye-measured hand-drawn images were found, but the result showed that even the hand-drawn blueprint can be converted into the 3D model without knowledge of 3D modeling by using the proposed MineLoC method. The 3D printable file converted results and the actual printed outputs are shown in Figure 3.

Accuracy was calculated by overlapping the models and counting the area of non-overlapping regions against the total area (defined by the rectangular base of the blueprint). The area was counted by counting the number of pixels of each region in top view.

First, to calculate an area error rate, the ratio between the non-overlapping (error) region and the total region was calculated. Then, the area accuracy was calculated as below:

(1) $$\left(\mathrm{Area}\;\mathrm{Accuracy}\right)=1-\left(\mathrm{Area}\;\mathrm{Error}\;\mathrm{Rate}\right)=1-\frac{\left(\mathrm{Error}\;\mathrm{region}\right)}{\left(\mathrm{Total}\;\mathrm{region}\right)}=0.860$$

To convert area accuracy to length error, the following equation was used:

(2) $$\begin{array}{cc} \left(\mathrm{Length}\;\mathrm{Error}\right)= & \left(\mathrm{Length}\;\mathrm{Error}\;\mathrm{Rate}\right)\times \left(\mathrm{Diagonal}\;\mathrm{Length}\right)=\sqrt{1-\frac{\left(\mathrm{Error}\;\mathrm{region}\right)}{\left(\mathrm{Total}\;\mathrm{region}\right)}}\times 15.52\;\mathrm{mm}\\ & \;=1.133\;\mathrm{mm} \end{array}$$

The result showed that the area of the non-overlapping region (error) was 2965 pixels while the area of the total region is 21,100 pixels. The accuracy of 86% was achieved by the proposed method. Using smaller block size could improve accuracy at the cost of user convenience and rendering time, but for our purposes in this paper the current block size was adequate.

### 3.2. Bacterial Cell Culture Experiment with the Fabricated Lab-on-a-Chip

**Figure 4:**
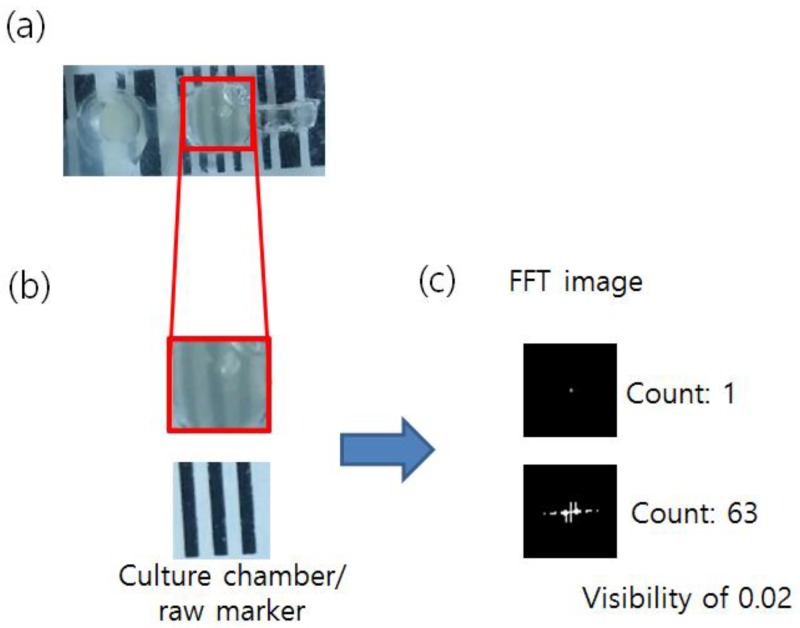
Bacterial growth detection experiment replicated on the fabricated PDMS devices: (**a**) bacterial cell culture on the PDMS device; (**b**) the blurred marker due to growth of the bacteria in the culture chamber of the PDMS device for 4 h (**up**) and the clear marker (**down**); and (**c**) the FFT spectrum count reduced due to the blurred marker. Count indicated the number of the nonzero pixels in the FFT image. The visibility was calculated by dividing the current count (1) with the count of the starting point (63).

While the copied PDMS template was originally designed for bacterial growth and its measurements, the authors have further cultured and analyzed the growth of bacteria using the PDMS chip, fabricated with the MineLoC designed mold. Moreover, the results were compared with previously reported data using a conventionally fabricated chip of the same chip design. Kim et al. [21,22], in the previous report, proposed a vision-based method to measure bacterial growth level in the culture vessel or micro/millifluidic devices. The striped patterned marker placed on the back side of the culture chamber and the camera was used to take the picture of the marker. The culture chamber with the high intensity of the bacteria covered the marker as the broth was blurred. The amount of the blurriness was measured by the fast Fourier transformation (FFT), and this value could also be converted to a more conventional growth measurement value using OD_600_. The same devices were generated using the proposed MineLoC method but with minor modifications to the design; culture chambers were enlarged for easier monitoring and for holding a larger volume of the culture broth to prevent drying by evaporation. The results are shown in Figure 4.

The results showed that the chip designed by the proposed MineLoC method can well reproduce the results from a device using the conventional fabrication process as shown in Figure S1. The count of Figure 4 was obtained by counting all nonzero pixels in the FFT image. The smaller count meant the broth with the higher turbidity blocked the observation of the patterned marker. The visibility was calculated by dividing the pixel count of the current state by the count before the culture started. The cell saturated broth (OD_600_ over 1.0) showed a visibility of 0.02, nearly zero, similar to the result from the conventional method, as shown in Figure S1 (visibility of 0.05). Thus, as shown by the reproducible growth measurement, the authors believe the PDMS device fabricated by the proposed method could be used as a cell culture chamber for growth analysis, similar to the conventional PDMS biosensors.

### 3.3. Generation of More Example Molds

**Figure 5:**
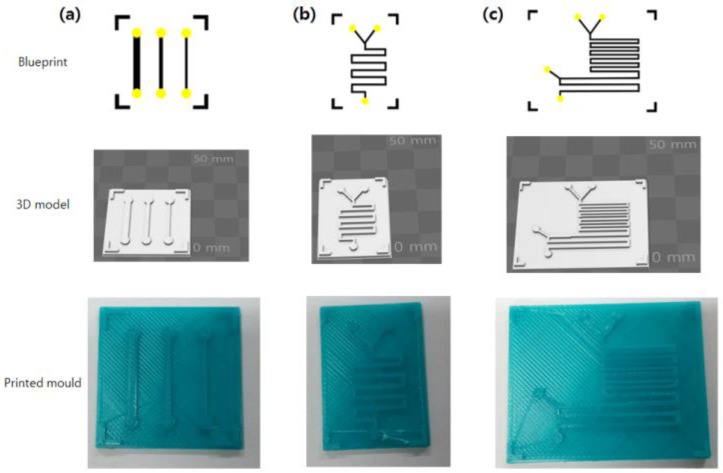
Blueprints, models, and printed molds of the other structures: (**a**) multi-width single channels for cell culture; (**b**) two liquid mixing channels; and (**c**) three liquid mixing channels.

Other Lab-on-a-Chip molds proposed by other researchers were also generated by MineLoC to show its further applications. The research done by Kim et al. [23] used a single channel device with multiple widths to culture bacterial cells and measure their biofilm formation ability in the microfluidic devices. Kitson et al. proposed the multiple liquid mixing and reaction channels for small scale chemical experiments [4]. The results are shown in Figure 5.

The PDMS molds for the various purposes can be easily and rapidly generated by simple blueprint drawings. Drawing for 3D printable model conversion takes only a few minutes with the proposed method. The mold fabrication time is also faster (though dependent on the size and quality) when comparing the speed of the 3D printer and soft lithography devices.

The authors tested different types of 3D printers to build the PDMS template: the Fused Decomposition Modeling (FDM) type (MakerBot Replicator 2X, MakerBot Industries LLC, Brooklyn, NY, USA) and the SLA printers from the other manufacturers. The FDM type printers have come into wide use as the printing materials are cheap and can be processed easily after printing. However, this type of 3D printer generates a lined pattern at the surface of the printed outputs due to the printing method. The PDMS generated by the FDM printed molds also has a rough surface due to the patterns of the master mold, which might be troublesome during the bonding process of the PDMS to the cover glass. A process smoothing the surface of the FDM printer output with acetone fumes was tested, but this did not fully resolve the issue. Since the FDM printer is one of the most easily accessible printers, the authors are considering this problem as a future work to increase the efficiency of the 3D-printer-based PDMS fabrication. Although the exact reason is yet to be determined, there were cases where PDMS did not fully cure on the master template generated by some SLA printers (Projet 1000, 3D Systems Inc., Rock Hill, SC, USA).

Recent reports show that the build orientation of the printing affects the time and cost for the manufacture [24]. The PDMS molds are generally flat planar objects with low height structures. The “backdown” orientation, which minimizes the height of the printing object (as shown in Figure 3), was used for the printing. This orientation helped stabilize manufacture by increasing aggregation between the printed objects and the 3D printer’s printing bed. Low height also aided in faster printing and prevented possible collapse during printing. The previous research also recommends the “backdown” orientation.

While the authors have not used the FDM printer for fabricating the mold, it is expected that the MineLoC method and the FDM printer can be used in the fabrication of the reactionwares proposed by Kitson et al. [4]. Some simple 2D structured chemical reactors were introduced which can be easily generated by colored pictograms, as shown in this research.

Resolution of the additive manufacturing is a current limitation factor of the protocol. The resolution is not necessarily an issue for milli-scale reactionware, but it can be problematic in micro-scale. When resolution becomes more important, usage of high-resolution or specialized printers in miniaturized printing could enhance the quality of resolution. The authors expect that the ongoing development in additive manufacturing and increasing supply rate of high-end 3D printers could soon eliminate these restrictions.

### 3.4. Comparisons with Other Modeling Software

Other modeling software also supports various modeling methods and functionalities. We investigated three types of software: web-based applications, voxel-based modeling tools, and software which supports collaborative design. For collaborative design, we specifically selected a software that supports synchronous co-modeling [25], which enables concurrent collaboration in modeling and modification process. BlocksCAD and TinkerCAD [26,27] are web-based modeling platforms that are accessible anywhere on most devices. However, in practice, such software is very slow in rendering the objects, and severely dependent on network performance. The voxel-based 3D block stacking modelers that are very easy to use are also available. However, 3D slash [28] software has limitations in the voxel number (128 per axis in the free version), inflexible user interface without user configurable features and restricted functionalities in the free version. MagicaVoxel [29] has a limitation in voxel number (126 per axis) and is unstable on the Windows operating system. The commercial software Onshape [30] supports the collaborative design but is not freely available. The key features are summarized in Table 1.

In voxel-based modeling software, including the proposed method, the accuracy can be improved by adapting smaller voxel size. However, such improvement is limited by the maximum number of voxels supported in the software. MagicaVoxel supports 126 voxels per axis, whereas 3D slash supports 128 voxels per axis in the free version and 512 voxels per axis in the paid version. In contrast, a Minecraft world can host 30,000,000 voxels for each of the horizontal axes and 256 voxels for the vertical axis. The proposed method successfully created up to 1000 × 1000 × 120 voxels, which is comparable to 3D slash (paid version). The proposed method also supports configurable voxel bounding box, which can be utilized to optimize the accuracy by the morphology of the intended model. Such features, as well as maximum resolution achievable when modeling the lab-on-a-Chip in Section 3.2, are summarized in Table 2.

Compared to other software tools, the proposed Minecraft-based method supports fast rendering, low difficulty in operation, and collaborative design without charging policy. Minecraft server program is freely available, and, while the client is proprietary, there exist open source ports which are compatible with “vanilla” servers [31]. As Minecraft is well known, widely sold and used software, the accessibility and user friendliness is much higher than other compared software. In addition, Minecraft server can be deployed on local machines, ensuring its performance regardless of the quality of Internet connection.

## 4. Conclusions and Future Work

MineLoC, an easy-to-use pipeline that generates directly printable 3D models of the PDMS curing master template, was developed and verified in this research. Utilizing a popular multi-player sandbox game “Minecraft”, MineLoC allows researchers, who are not trained to design 3D geometry or utilize 3D CAD software, to generate masters for LoC devices. Furthermore, the multiplayer feature of Minecraft enhance the collaborative design, which enables a more efficient and agile design process. The authors anticipate the tool will facilitate more use of 3D printer-based LoC fabrication, which greatly lowers the entry barrier in the field of Lab-on-a-Chip research.

Development of classic microfluidic structures to be used for the fabrication of PDMS devices into 3D-printed plastic materials is considered for further application. Various techniques, such as Christmas-tree structure [32], herringbone structure [33], and micro valves with multiplexed channels [34], have been developed to handle various experiments. Such pre-developed modules and structures could be included automatically into the game world model during the conversion of the 2D pictogram.

## Figures and Tables

**Table 1:** Summarized features of the other modeling software and the proposed method.

	BlocksCAD	TinkerCAD	3D Slash	MagicaVoxel	Onshape	Proposed
Price policy	Free	Free	Free ^2^	Free	Not free ^3^	Free (server)
Rendering	Slow	Slow	Fast	Fast	Fast	Fast
Difficulty	Medium	Medium	Low	Low	Very High	Low
Co-modeling	N/A ^1^	N/A	N/A	N/A	Available	Available

^1^ Not available. ^2^ With restricted functionalities. ^3^ Free for Open-source projects only.

**Table 2:** Accuracy comparison between other voxel-based software and the proposed method.

	MagicaVoxel	3D Slash (Free)	3D Slash (Paid)	Proposed
Voxel Limit	126 × 126 × 126	128 × 128 × 128	512 × 512 × 512	1000 × 1000 × 120 ^1^
Configurable Bounding Box	N/A	N/A	N/A	Available with 256 voxels height limit
Resolution ^2^	0.302 mm	0.297 mm	0.0742 mm	0.0500 mm ^1^

^1^ Higher accuracy achievable by optimizing bounding box. ^2^ When modeling the lab-on-a-chip in Section 3.2.

## Supplementary Materials

The following are available online at http://www.mdpi.com/1424-8220/18/6/1896/s1, Figure S1: The PDMS devices used for the bacterial culture using chips fabricate by conventional method [21]. (a) The chip on the marker before the incubation (up) and after the incubation (down). (b) Magnified view of the region of interests. (c) Reduced FFT spectrum size with growth of bacteria.

## Appendix A. Detailed Steps of Deploying MineLoC Server

1. Install Minecraft 1.9.4, MineWays (http://www.realtimerendering.com/erich/minecraft/public/mineways/), and MeshLab (http://meshlab.sourceforge.net)
2. Download CraftBukkit server (https://www.getbukkit.org/craftbukkit.html). Version 1.9.4 was tested.
3. Write “java -Xms1024M -Xmx1024M -jar craftbukkit.jar -o false PAUSE” in the notepad and save it as a run.cmd (for Windows)
4. Open generated eula.txt file (generated when run.cmd is executed for the first time) and change eula option to true.
5. Use terminal to execute run.cmd. Check access to the server.
6. Open server.properties file. It is recommended to use the FLAT world setting (level-type) with no npcs, mobs, or animal spawn (spawn-monsters, spawn-animals, and spawn-npcs options). Change gamemode=1 (creative mode) for easier manipulation of the world. Change other options if necessary.
7. Download WorldEdit form https://dev.bukkit.org/bukkit-plugins/worldedit/. WorldEdit-bukkit 6.1.2 was tested.
8. Extract *.jar file and copy to the /plugin folder generated by the server. Restart the server and check generation of /plugin/WorldEdit folder.
9. Generate /plugin/WorldEdit/craftscript folder and /plugin/WorldEdit/drawings folder for script execution.
10. Save *.js script in craftscript folder and images in drawing folder.
11. Download Rhino JavaScript engine Ver. 1.7.7.1 (https://developer.mozilla.org/en-US/docs/Mozilla/Projects/Rhino/Download_Rhino)
12. Extract rhino-*.jar from the /lib folder and copy under /WorldEdit folder and change the name to js.jar.
13. Restart the server and use the op <username> command to obtain permission.
14. Test WorldEdit scripts. LoC generation script used in this research is available at (Github): https://github.com/W5-KAIST/MineLoc. “drawloc.js” is the currently tested version converting red, blue, yellow, and black colors to block structures.

## Appendix B. Detailed Steps of Operating MineLoC in the Virtual World

1. Execute Minecraft and enter the MineLoC server by entering multiplayer mode.
2. The server command usage was “//cs drawloc.js locimage.png”.
3. Check the generated structure and apply modifications if necessary (for stability of the output, it is recommended to stop the server first to save the changes).
4. Run MineWays and clicked File → Open World → Find your world menu
5. “World Data” is located at /world/level.dat file of the Minecraft server.
6. Find the required build model and draw a square region with a right click.
7. Save it as a Shapeways VRMW file (*.wrl) format.
8. Adjust Height value, 3D printing options and erased superhollow and hollow out bottom option.
9. Open the MeshLab and imported generate *.wrl file by clicking File → Import Mesh option.
10. Check the model and export it by clicking File → Export Mesh As menu.
11. Click *.stl option to get STL file for the 3D printing.
