# Supplementary material for: MineLoC: A Rapid Production of Lab-on-a-Chip Biosensors Using 3D Printer and the Sandbox Game, Minecraft
Source: Sensors (Basel). 2018 Jun 10;18(6):1896. doi: 10.3390/s18061896 (PMC6021845; doi:10.3390/s18061896)
Supplement: Supplementary file 1 [file sensors-18-01896-s001.pdf]

Supplementary Material

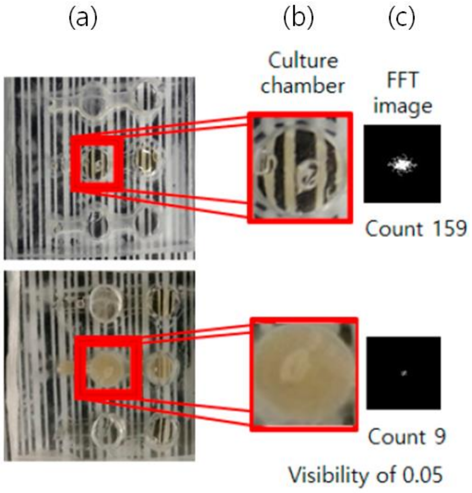

Figure S1. The PDMS devices used for the bacterial culture using chips fabricate by conventional method [1]. (a) The chip on the marker before the incubation (up) and after the incubation (down). (b) Magnified view of the region of interests. (c) Reduced FFT spectrum size with growth of bacteria.

References

1. Kim, K.; Choi, D.; Lim, H.; Kim, H.; Jeon, J. Vision Marker-Based In Situ Examination of Bacterial Growth in Liquid Culture Media. *Sensors* **2016**, *16*, 2179.
